# T-Pattern Detection and Analysis (TPA) With THEME^TM^: A Mixed Methods Approach

**DOI:** 10.3389/fpsyg.2019.02663

**Published:** 2020-01-10

**Authors:** Magnus S. Magnusson

**Affiliations:** Human Behavior Laboratory, School of Health Sciences, University of Iceland, Reykjavik, Iceland

**Keywords:** behavior, interaction, T-pattern, ethology, pattern detection, fractal, software

## Abstract

This work, which was started in the early 1970s, was inspired by social interaction analysis based on direct observation and careful coding of behaviors according to a list of behavioral (mostly ethological) categories, especially the ethological work of N. Tinbergen, K. Lorenz, and K. von Frisch, for which they shared a Nobel Prize in 1973 in Medicine or Physiology but also H. Montagner’s ethological analyses of interactions in social insects and children. S. Duncan’s psychological and linguistic research on turn-taking in human interactions provided great inspiration, and so did Chomsky’s work on syntactic structure and Skinner’s probabilistic real-time functional analysis and their consequent debate. A hypothesis concerning numerous kinds of temporal and spatial natural and especially biological structures, the T-pattern is a hierarchical self-similar fractal-like structure that recurs with significant translational symmetry on a single discrete dimension, initially real time. It also points to profound self-similarity across many levels of biological spatio-temporal organization, as it seems characteristic of molecular structures such as genes and a multitude of recurrent motives on DNA and its 3D generalization corresponding to (3D) folded proteins. Developed initially to facilitate empirical analysis, the T-pattern and its detection algorithms were first presented in AI (Magnusson, 1981) and Applied Statistics (Magnusson, 1983) through THEME (3 k Fortran IV) software using an evolution algorithm. It is now over 300 k lines of code, runs under Windows, and, more recently, uses parallel processing for increased speed. This has allowed abundant detection of hidden structure in numerous kinds of biological phenomena at highly varied scales, from human behavior at timescales of days (Hirschenhauser et al., 2002; Hirschenhauser and Frigerio, 2005) to interactions of many individual neurons simultaneously registered at a temporal resolution of 10^–6^ s in neuronal networks in rat brains to ongoing work on T-patterns in DNA molecules at a spatial nano-scale. T-pattern detection and analysis (TPA) thus mix qualitative and quantitative analyses, as T-patterns themselves are artificial categories composed of recurring coding categories with special real-scale statistical relations between their instances. After their detection, T-patterns are thus analyzed much as are other behavioral categories.

## Introduction

As a Mixed Methods approach, T-pattern Analysis (TPA) passes repeatedly between qualitative and quantitative analyses, from data collection logging the occurrences of qualities (categories) and their real-time (quantitative) locations resulting in time-stamped data, here T-data, to the detection of T-patterns (qualities) defined below, typically followed by both qualitative and quantitative analyses of the detected patterns. TPA is primarily intended for structural exploration but has most often been combined with standard statistical methods for the detection of the effects of external (experimental) variables.

The initial inspiration for the development of this approach was the ethological work of Niko Tinbergen, for which he, in 1973, shared the Nobel Prize in Medicine or Physiology with Karl von Frisch and Konrad Lorenz. The present project, which began in the early 1970s and has led to TPA, was influenced more specifically from many other directions including ethological and human interaction research (for example, Tinbergen, 1963; Eibl-Eibesfeldt, 1970; Montagner, 1971, 2012; Dawkins, 1976; Duncan and Fiske, 1977), linguistics (for example, Chomsky, 1957), and also radical behaviorism (for example, Skinner, 1969), all focusing on recurrent hierarchical and syntactically constrained temporal sequences, patterns, or contingencies. All were concerned with non-random recurrent synchronic and/or sequential temporal patterns of behaviors that were often themselves such patterns. For example, in verbal behavior, common phrases are composed of common combinations of words, which are combinations of syllables that are themselves sequential patterns of phonemes, or letters in the case of written language. Some such word combinations occur as parts of recurring interactive verbal and/or non-verbal behavior patterns, where different individuals react to each other with characteristic (predictable) timing constraints. Constraints of order and relative timing include those characteristic of melodies and numerous verbal and/or non-verbal routines, ceremonies, and rituals of everyday life, including some widely recurring texts, some even called holy.

Exploration of statistical and AI computer methods indicated that new, more specifically tailored methods were needed for the discovery and analysis of hidden structure in behavior and possibly some other biological phenomena (Magnusson, 2004). The definition of a T-pattern below thus attempts to integrate aspects of known recurrent behavioral patterns in a formal pattern definition and algorithms for the computational detection of some otherwise hidden patterns.

This initially led to the definition and detection of “temporal configurations” as a kind of “artificial category”, first presented in an AI and Applied Statistics (Magnusson, 1981, 1983, 1996, 2000) and now called T-patterns, which, with gradually added structural types, now forms the T-system for the structural analysis of behavior, interactions, and other mostly biological phenomena. The aim has been to obtain new objective, quantitative, and structural (qualitative) bio-mathematical insights into the structure of behavior through the formulation of hypothetical mathematical pattern types to be evaluated with corresponding detection algorithms in the dedicated software THEME^TM^, which also provides specially developed diagrams for the visualization of T-data and T-patterns such as are found in this paper.

The parallel processing available on multicore PC processors now provides increased speed, facilitating TPA application and further development. Theme is now Windows software, including a free academic version, which can be downloaded from www.patternvision.com.

T-pattern detection and analysis has been applied in a number of research areas concerned with very different time scales, from 10^–6^s in neuronal interactions (Nicol et al., 2005, 2015) to days and even years (Anolli et al., 2005; Magnusson, 2005, 2006, 2016, 2017, 2018; Casarrubea et al., 2015, 2018; Magnusson et al., 2016).

As the T-system concepts, algorithms, and applications have been widely published, this paper is concerned with other aspects such as the meaning of T-patterns and their (biological) relevance for the discovery and understanding of behavior and related biological phenomena such as T-patterned strings, called T-strings, the omnipresent texts in recent human mass societies.

## Methods

The following sections describe the type of data referred to by all T-system definitions, followed by an essential description of the T-pattern and its corresponding detection algorithm as implemented in the Theme software.

### T-Data

**FIGURE 1:**
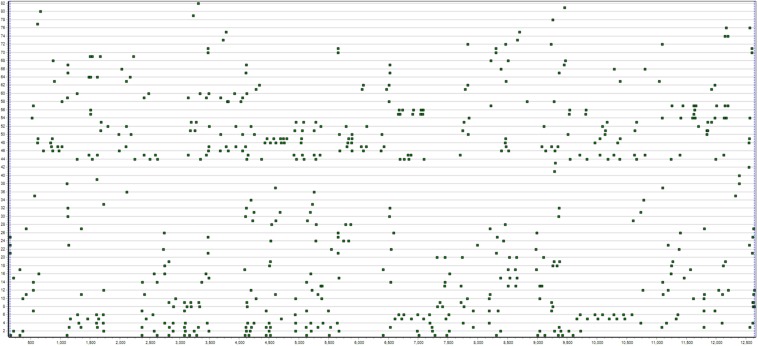
This figure shows T-data of a 13.5 min children’s free dyadic object play interaction where two 5-year-old girls take turns watching pictures in a picture-viewer. Each line of points represents the start or stop moments for a particular behavior for one or the other. In this image, the 82 series are sorted by actor (child) and frequency.

All T-system definitions and TPA algorithms refer exclusively to a type of data, here called T-data (Figure 1) consisting of one or more sets of discrete (occurrence) point series, each set (here also called a sample) occurring within a continuous observation period. The collection of T-data itself uses a mixed method, as qualitative categories (here called event-types) with their real-time (quantitative) occurrence points are recorded. The data are stored in two-column.txt files; [time tab event-type], which are the required input to all Theme processing. Initially, each sample is stored in a separate file, but all can optionally be concatenated and analyzed together as a single multi-sample file. Patterns may thus be detected in each sample file separately or independently or across all samples in a single multi-sample file. The baseline probabilities used in pattern detection are calculated independently for each file, whether a single-sample file or a multi-sample file and will thus vary for some event-types. The setting of a search parameter, such as the minimum number of occurrences, must also take into account that while no pattern may occur that often in a single-sample file, it may occur much more often across all the samples in a multi-sample file. *Theme project* refers to all samples analyzed together, whether in a multi-sample file or separately (see the Theme manual for details).

Visualization of raw T-data is provided in Theme for overview of the data and to help identify coding errors.

### The T-Pattern

Here only an essential description is presented of the hierarchical, multiordinal, and self-similar T-pattern type and the essential corresponding detection algorithm implemented in Theme.

A T-pattern, Q, comprises m ordered components, X_1__..__m_, recurring in a single discrete dimension, where each component is a T-data category (or pattern primitive, called event-type) or a T-pattern:

Q:X1[d1,d2]1X2[d1,d2]2…Xi[d1,d2]iXi+1…  X[d,1d]2m-1Xm-1 m(m=length)

where, over the n occurrences of the pattern within T-data, each of the distances X_i_ to X_i+__1_ is significantly similar relative to a zero hypothesis of independent random distribution of each component. Each of these distances thus varies within a different one of the m-1 intervals, called critical intervals, [d_1_, d_2_]_i__;_ *i* = 1..(m−1); where 0 ≤ d_1_ ≤ d_2_.

For special situations, this definition is restricted in various ways depending on the type of data and detection purposes. The current binary-tree bottom-up search algorithm of the Theme software relies on finding in T-data at least one pair of series related by a critical interval and then adding its occurrence series to the T-data and thus including it in the continued search for more pairs and possibly pairs of pairs etc. This binary tree approach has allowed detection of numerous and often quite complex T-patterns, but as will be exemplified below, this does not guarantee the detection of all possible T-patterns in the data, which might require using a trinary or higher tree.

T-patterns can be called recurrent hierarchical and multi-ordinal or, in more modern language, self-similar statistical pseudo fractal entities (objects) characterized by significant translation symmetry between their occurrences.

### T-Pattern Detection Algorithm

Restricting the T-pattern definition above for detection purposes, any T-pattern Q: X_1_ X_2_..X_*m*_ can be split top-down recursively into a pair of shorter ones related by a corresponding critical interval, CI:

Q=Q[d,1d]2LeftQRight

Recursively, Q_Left_ and Q_Right_ can each be split until the full T-pattern is expressed as the 1..m terminals X_1_..X_m_ of a binary-tree of non-terminal critical interval relations.

Detection works in the opposite direction of the splitting above, that is, bottom-up, beginning with the series in T-Data and using special algorithms for critical interval detection, pattern construction, and pattern completeness competition (evolution algorithm), where redundant detections of the same underlying patterns are ignored. Theme then provides two types of statistical Monte Carlo validation.

### Statistical Validation

**FIGURE 2:**
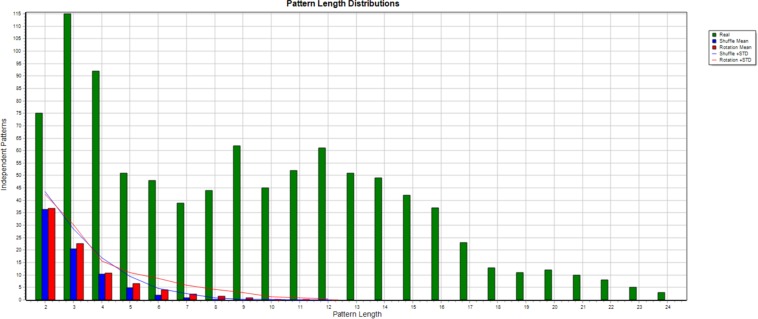
This figure shows the result of a Monte Carlo (50 randomizations) comparison. The number of different T-patterns of each length detected in the initial data is shown in green, while the averages for shuffling and rotation are shown in blue and red, and the blue and red lines add one standard deviation to each.

When numerous significance tests are calculated, many may be positive even when the data is random, so it is necessary to evaluate to what extent this explains the detection of T-patterns in a dataset for given search parameter values. Two methods are provided in Theme, each using a different type of randomization, T-shuffling, or T-rotation. Under T-shuffling, each of the series in T-data is replaced with a series of random numbers within the observation interval, [1, T]. Under T-rotation, each series, t_i_, is shifted by a new random value, dt, where 0 < dt < T, so t_i_ = [(t_i_ + dt) mod T]. Each method repeatedly randomizes the data, searches for T-patterns, and stores the number of different patterns of each length found. Finally, the averages over all the randomizations are calculated and compared with the number detected in the original data. The differences found for each pattern length are usually far greater than required for significance (Figure 2).

### Visualization

**FIGURE 3:**
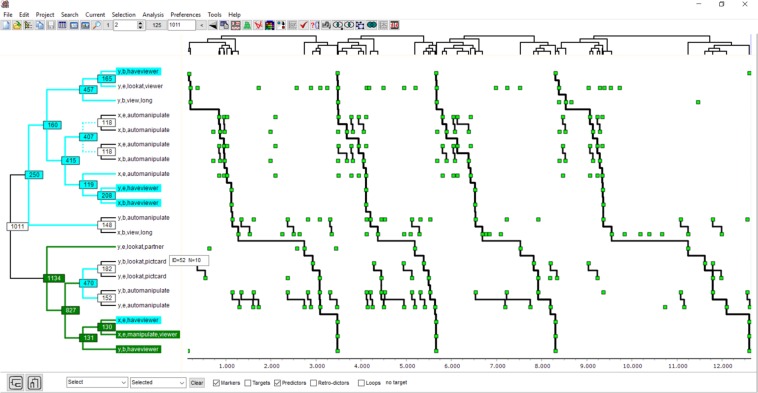
This figure shows one T-pattern detected in the T-data shown in Figure 1. The clustering diagram on the left shows how points in the series on the right are connected level-by-level to form the four occurrences of the complete pattern. *x* and *y* in the labels represent the two girls, and b or e stand for beginnings or ends.

Visualization using a specially developed type of interactive diagram (Figure 3) primarily allows qualitative analysis but also provides some quantitative information. Each diagram shows all the occurrence (point) series in T-data that are involved in the pattern and all the bottom-up, level-by-level connections of points to form the full pattern. Theme software allows various interactive visualizations of T-patterns, other T-system structures, and their relations that cannot be presented in a paper but can be freely explored using the (free) academic version available at www.patternvision.com.

The following T-pattern diagram concerns the dyadic interaction for which the T-pattern model, algorithm, and software were first developed (Figure 3). Even though, over decades, the approximately 13.5 min interaction between two 5-year-old girls has been coded repeatedly and then searched for T-patterns, there is often something new to be noticed, as no single T-pattern captures all that is happening but still adds some new insight.

### Restricted T-Patterns

Special T-pattern types are defined by specially restricted critical intervals such as the fast critical interval [0, d_2_] defining the univariate T-pattern type, called T-bursts, which are sudden increases in frequency in a single T-data occurrence (point) series. T-bursts may occur alone and/or as a branch of other T-bursts or T-patterns. A common characteristic of T-bursts is to sometimes greatly improve the prediction of other behaviors.

### T-Patterns and Cyclicity

While the definition and detection of T-patterns are not based on cyclical occurrence, and just two occurrences of a T-pattern may allow detection, T-patterns often occur cyclically and may thus bring to light cyclical relations between T-data series where cyclicity is not present in any of the single series (Magnusson, 1989).

### [1, 1] Restricted T-Patterns

Another restricted T-pattern type has the fixed critical interval [1, 1] and is used for some TPA of text or molecular sequences (DNA and proteins, in preparation) notably for the detection of recurrent continuous strings such as DNA codons or words within texts (see below).

### The T-System

Starting with the T-pattern and its univariate version, T-burst, other structural aspects have been added to the T-System, including T-Markers, T-Predictors, T-Retrodictors (Magnusson, 2017), and T-packets with ±T-Associates as well as T-Composition have been described elsewhere (Magnusson, 2005, 2006, 2016, 2017).

## Qualitative and Quantitative Analysis of Detected T-Patterns

After detection, a new set of analysis tools is used to extract qualitative and quantitative information of interest from the set of detected patterns. In addition to the detection and visualization of T-patterns and other T-system types, the Theme software provides pattern selection features and output of corresponding tables for quantitative analyses using standard statistical methods and tools.

### Structural Qualitative Aspects

Most of the qualitative analyses concern the implication and structural positions of coded behaviors or T-patterns of special interest within more complex T-patterns as recurring context. Program features allow the selection of patterns, including any or all of specified behaviors and, optionally, in a specified order. A list of all coded behaviors occurring in selected patterns is also available, as well as at what hierarchical pattern levels each first appeared. As the coded behaviors usually specify the actor (agent, individual, group, etc.) of the behavior, detected multi-actor patterns suggesting interaction and/or synchronization can also be selected.

**FIGURE 4:**
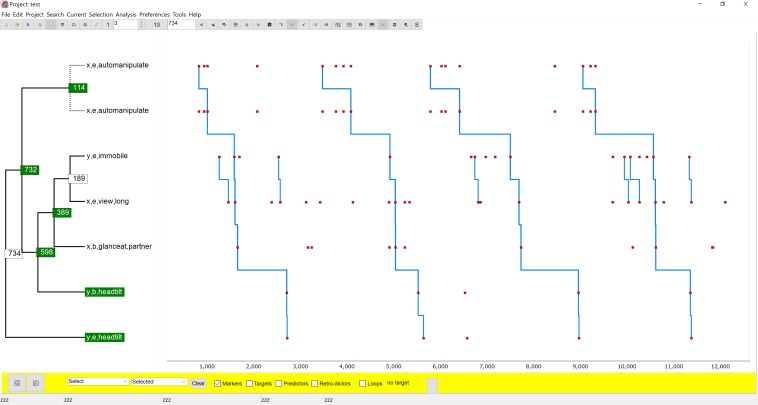
This figure shows a T-pattern different from Figure 3 but detected in the same T-data showing strong relations between the behaviors of each child related to the transfer of the toy. It is characteristic of girl x that when she is waiting to get the toy again, she repeatedly fiddles with something, easily perceived as a sign of impatience. Freezing (immobile) only occurs in y and only when she is waiting for the toy. Finally, the head tilt, often associated with begging, only occurs in y and then only when she is waiting.

Figure 4 is an example of TPA used for qualitative structural exploration. It is of interest here as it captures, in a single T-pattern, the relations between a number of behaviors implicated in each of the four dyadic object transfers described above and underlines that a single T-pattern usually does not capture all the T-patterning found in a T-data set and combining information from two or more may provide better insight.

**FIGURE 5:**
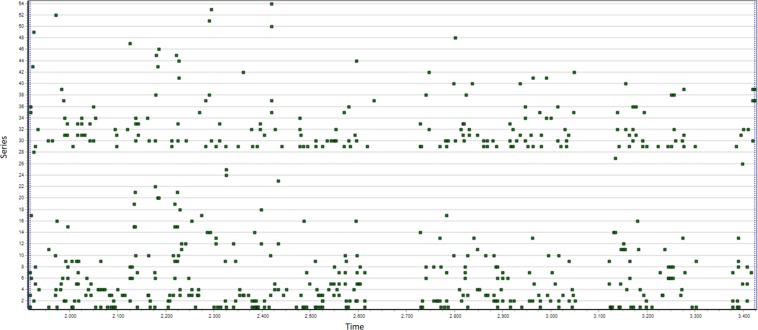
This figure shows T-data of one problem solving dyad, where the two children (e and n) attempt to solve together a puzzle with three consecutive stages.

**FIGURE 6:**
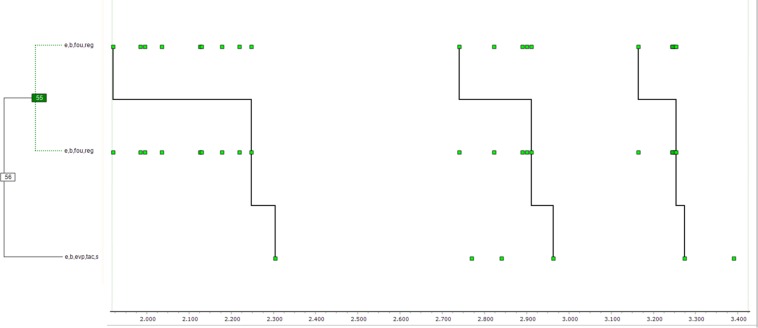
This figure highlights bursts, here providing a rule (reg) for the solution at each stage, followed by a positive evaluation of progress (evp) by one of the two, talking to herself (s = soliloquy). Each of the behaviors in the burst clearly has little predictive behavior, but an occurrence of the burst each time predicts the positive evaluation.

Figure 6 recently detected in data (Figure 5) from a previous project (Magnusson and Beaudichon, 1997) concerning children’s dyadic problem-solving interactions, is the T-pattern in Figure 6. The puzzle had three stages, and this particular dyad was the slowest to reach completion and was therefore expected to be relatively unstructured. The T-burst within the T-pattern shown in Figure 6 is different from those shown elsewhere (for example, Magnusson, 2016, 2017) in that each of its occurrences is of relatively long duration but still shows increased predictive power of a T-burst relative to single occurrences of its elements.

### Quantitative Analyses

Quantitative analysis of a set of detected T-patterns usually concerns their number of occurrences, number of components, hierarchical levels, and number of actors and switches between them within the pattern. When subsets of the samples have been collected under different experimental conditions, such parameters have often allowed the discovery of effects of independent (external or background or experimental) variables. Numerous applications rely on this, as can be seen in a recent comprehensive review (Casarrubea et al., 2015).

## Spatial T-Patterns and Self-Similarity

The T-pattern and TPA were created primarily to help decipher the little known “languages” of non-verbal interactions among various organisms through the use of artificial means, here computational algorithms, to discover hidden or non-obvious patterns in real-time streams of behavior and, consequently, to try to understand their function or meaning and sometimes diagnostic value for distinguishing the behavioral profiles of individuals, groups, or (experimental) conditions.

It was in continuation of a number of bilateral collaborations regarding such interaction research that in 1995, seven European universities signed a collaboration convention around TPA entitled Methodology for the Analysis of Social Interaction. This growing network now includes 32 universities in Europe and the United States.

However, recent discoveries in cell biology, genetics, and proteomics have drawn attention to spatial T-patterns in physical strings and analogies between, on one hand, the purely informational physical strings (DNA) existing in all biological cells since billions of years ago, holding the blueprints for the numerous types of specialized citizens in the mass societies of proteins (now sometimes called “Cell Cities”) and, on the other hand, texts, which have appeared in a biological eye-blink, with analogous blueprints for specialized individuals and now influencing practically all human behavior, changing naked apes into string-enabled and string-controlled mass-social citizens. This view of the modern mass-social context of human interaction has only recently become possible thanks to new technology and discoveries in cell biology including genetics and proteomics, but such self-similarity across many orders of magnitude seems to underline the possible broad relevance of the T-pattern model and TPA at different levels of temporal and spatial biological organization. The latest addition to the T-system is thus physical strings containing spatial T-patterns, called T-strings, exemplified primarily by DNA and texts. In this light, some TPA of texts (and DNA) has therefore begun, with the first results now appearing.

### Detecting Words in Text as T-Patterns and Their Meaning

For literate speakers of a language, its words and various word combinations are usually obvious in both speech and text and have a fairly clear meaning that is hidden to non-speakers. The same is true for many detected T-patterns in behavior and interactions, which, even after detection, may remain invisible to the naked eye or have no obvious “meaning.”

**FIGURE 7:**
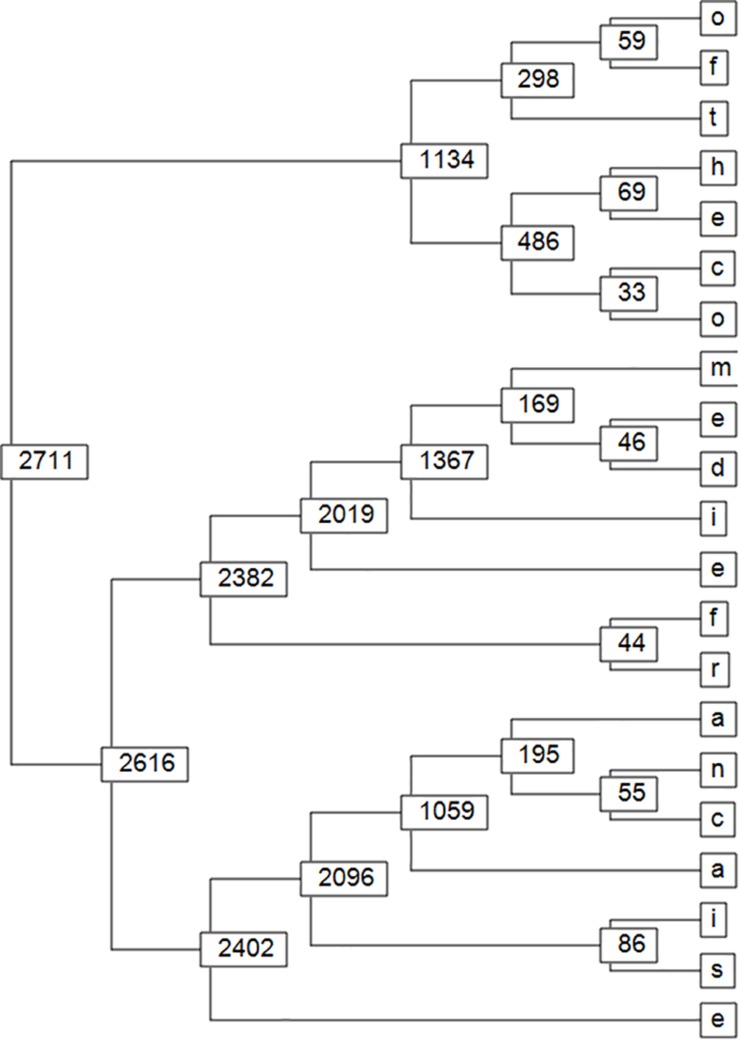
This figure shows the gradual bottom-up detection, level-by-level, of a long recurrent T-pattern of letters: “ofthecomediefrancaise” even if occurring only twice in the short text where word separations (blanks) were removed.

**FIGURE 8:**
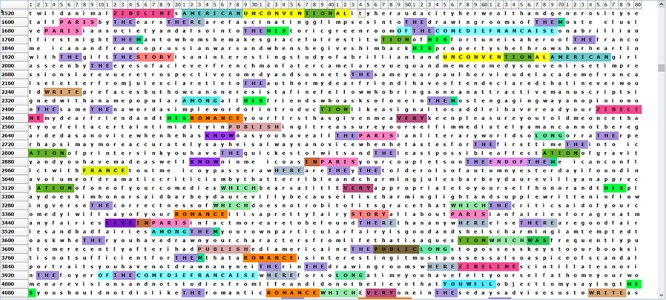
This figure shows some of the many recurrent letter T-patterns in a short text. It shows that some such patterns may not be easily given a particular meaning, as some common letter combinations often occur within words or as word endings, for example, “tion”, while the common string “the” can occur alone or as a part of a longer word.

A search for [1, 1] restricted T-patterns was made in a short text of 10103 letters (called “Zibeline” and randomly found and downloaded from the internet) but with word separators removed (i.e., blanks deleted) to see if TPA using the limiting binary tree approach (rather than trinary trees or higher) can detect recurrent words and possibly word combinations as T-patterns. Where all the words are known *a priori*, this can also help answer questions about limitations of the algorithm and the meaning of T-patterns (Figures 7, 8).

## Discussion

Important limitations of the current T-pattern should be mentioned here. While they need to be addressed, the current algorithm led to abundant detections of patterns where only minimal patterning had been found before using other methods, thus shifting the focus and limited programing resources to visualizing and analyzing the many detected patterns.

However, the following limitations exist and are being addressed.

First is the limitation caused by the exclusive use of binary-tree detection, which may overlook many T-patterns detectable with a higher-order tree. Recent searches for [1, 1] restricted T-patterns in text as occurrences of letters show that some words cannot be detected for this reason as, for example, some words include no significant pairs of letters, while, as a whole, they are highly significant T-patterns and would thus be detectable with higher-order trees.

Another major limitation is that the significance level is decided by the user rather than being detected by a special algorithm, long since among the top priorities in TPA development. A guideline for deciding on a significance level is briefly described elsewhere (Magnusson, 2000). This involves a kind of bootstrapping, that is, trying out different levels optimizing different aspects, depending on the main interest of the study, such as the maximum or average lengths or levels of detected patterns while giving good Monte Carlo results. An as yet unexplained observation is that significance levels near 0.005 are often found to be the best, which is why that value is the default value in Theme, to be modified as needed by the user.

A further limitation is the lack of consideration of substitutes or alternatives, such as when different components may occur at some position in a pattern, as each variant can only be detected as a different pattern and only if the variants occur the required number of times. This has become a more pressing concern because of a pilot study of [1, 1] restricted T-patterns in proteins testing whether some known patterns constitute significant T-patterns. All were found to be highly significant, but testing a large number (all) of such known patterns is in preparation. Most would not be detectable with the current algorithm, as their definitions typically include alternatives at some positions.

Algorithmic solutions for each of these limitations are in preparation and to be implemented in the next Theme version.

Some notes follow concerning the apparent biological interest and validity of the T-pattern and the derived T-string concept.

This work is rooted in Human Ethology, where a central theme is respecting the special characteristics of each species. But ethology, with its main focus initially on animal research, was not well prepared for the human kind of language and even less its written form, text, a recent and powerful kind of external memory appearing in a biological eye-blink but without which modern human behavior can hardly be understood. It was even less prepared for recent fractal mathematics highlighting self-similarity over numerous scales in the increasingly known universe (Rees, 1999; Baryshev and Teerikorpi, 2002; Kautz, 2011).

Highly structured mass societies of, for example, >10,000 individuals are very rare and in non-human animals are found only in insects. While external memory (texts) is essential in modern humans and in cells (DNA) for the specialization of their (human vs. protein) citizens, in insect societies, specialization is achieved very differently (Hölldobler and Wilson, 2008). Obviously, there is no direct or simple evolutionary path between the internal workings of biological cells and human mass societies, but, as underlined by Konrad Lorenz in his inaugural Nobel Lecture (Lorenz, 1974), analogy is a valuable source of knowledge in ethology, and here it provides a new perspective on the human situation where dramatic changes are taking place, not in genes, but in lifestyle.

While the discovery of the biological cell itself is so recent and that of DNA, the ribosome, and the RNA world even more so, “Cell City” now appears an attractive model for human behavior in modern human mass societies. Purely informational strings (DNA) suddenly became essential and omnipresent for the specialization, enabling, and control of the numerous and varied protein citizens. The RNA world became a DNA world of very complex cells, and bio-mathematical self-similarity was reached as the illiterate world became a purely informational text-based mass-social world. Both text and DNA seem to be T-strings to a large extent, suggesting spatio-temporal self-similarity over numerous levels of biological organization from nano to human scales (Magnusson, 2005, 2009, 2016), also indicating possible broader applicability of TPA.

## Data Availability Statement

The datasets analyzed in this manuscript are not publicly available. Requests to access the datasets should be directed to corresponding author.

## Ethics Statement

Ethical review and approval was not required for the study on human participants in accordance with the local legislation and institutional requirements. Written informed consent from the participants’ legal guardian/next of kin was not required to participate in this study in accordance with the national legislation and the institutional requirements.

## Author Contributions

The author confirms being the sole contributor of this work and has approved it for publication.

## Conflict of Interest

The author is the sole author and copyright owner of the THEME software and principal owner and COB of PatternVision Ltd. (www.patternvision.com) responsible for the marketing of the THEME^TM^ software.
